# Risk factors for acute kidney injury in patients hospitalized with COVID-19

**DOI:** 10.1590/2175-8239-JBN-2023-0056en

**Published:** 2023-12-08

**Authors:** Carolina Larrarte Arenas, Andrés Camilo Prieto Forero, Diana Carolina Vargas Ángel, Pedro Manuel Rincón López, Lizeth Vanessa Gómez Diaz, Diana Katherine Navas Aguilar, Henry Camilo Morera Yate

**Affiliations:** 1 RCS Hospital Militar Central, Bogotá, Colombia.

**Keywords:** Acute Kidney Injury, Corona­virus Infections, COVID-19, SARS-CoV-2, Risk Factors, Injúria Renal Aguda, Infecções por Coronavírus, COVID-19, SARS-CoV-2, Fatores de Risco

## Abstract

**Introduction:**

Acute kidney injury (AKI) occurs frequently in COVID-19 patients and is associated with greater morbidity and mortality. Knowing the risks of AKI allows for identification, prevention, and timely treatment. This study aimed to identify the risk factors associated with AKI in hospitalized patients.

**Methods:**

A descriptive, retrospective, cross-sectional, and analytical component study of adult patients hospitalized with COVID-19 from March 1 to December 31, 2020 was carried out. AKI was defined by the creatinine criteria of the KDIGO-AKI guidelines. Information, regarding risk factors, was obtained from electronic medical records.

**Results:**

Out of the 934 patients, 42.93% developed AKI, 60.59% KDIGO-1, and 9.9% required renal replacement therapy. Patients with AKI had longer hospital stay, higher mortality, and required more intensive care unit (ICU) admission, mechanical ventilation, and vasopressor support. Multivariate analysis showed that age (OR 1.03; 95% CI 1.02–1.04), male sex (OR 2.13; 95% CI 1.49–3.04), diabetes mellitus (DM) (OR 1.55; 95% CI 1.04–2.32), chronic kidney disease (CKD) (OR 2.07; 95% CI 1.06–4.04), C-reactive protein (CRP) (OR 1.02; 95% CI 1.00–1.03), ICU admission (OR 1.81; 95% CI 1.04–3.16), and vasopressor support (OR 7.46; 95% CI 3.34–16.64) were risk factors for AKI, and that bicarbonate (OR 0.89; 95% CI 0.84–0.94) and partial pressure arterial oxygen/inspired oxygen fraction index (OR 0.99; 95% CI 0.98–0.99) could be protective factors.

**Conclusions:**

A high frequency of AKI was documented in COVID-19 patients, with several predictors: age, male sex, DM, CKD, CRP, ICU admission, and vasopressor support. AKI occurred more frequently in patients with higher disease severity and was associated with higher mortality and worse outcomes.

## RESUMO

**Introdução:**

Lesão renal aguda (LRA) ocorre frequentemente em pacientes com COVID-19 e associa-se a maior morbidade e mortalidade. Conhecer riscos da LRA permite a identificação, prevenção e tratamento oportuno. Este estudo teve como objetivo identificar fatores de risco associados à LRA em pacientes hospitalizados.

**Métodos:**

Realizou-se estudo descritivo, retrospectivo, transversal e de componente analítico de pacientes adultos hospitalizados com COVID-19 de 1º de março a 31 de dezembro, 2020. Definiu-se a LRA pelos critérios de creatinina das diretrizes KDIGO-LRA. Informações sobre fatores de risco foram obtidas de prontuários eletrônicos.

**Resultados:**

Dos 934 pacientes, 42,93% desenvolveram LRA, 60,59% KDIGO-1 e 9,9% necessitaram de terapia renal substitutiva. Pacientes com LRA apresentaram maior tempo de internação, maior mortalidade e necessitaram de mais internações em UTIs, ventilação mecânica e suporte vasopressor. A análise multivariada mostrou que idade (OR 1,03; IC 95% 1,02–1,04), sexo masculino (OR 2,13; IC 95% 1,49–3,04), diabetes mellitus (DM) (OR 1,55; IC 95% 1,04–2,32), doença renal crônica (DRC) (OR 2,07; IC 95% 1,06–4,04), proteína C reativa (PCR) (OR 1,02; IC 95% 1,00–1,03), admissão em UTI (OR 1,81; IC 95% 1,04–3,16) e suporte vasopressor (OR 7,46; IC 95% 3,34–16,64) foram fatores de risco para LRA, e que bicarbonato (OR 0,89; IC 95% 0,84–0,94) e índice de pressão parcial de oxigênio arterial/fração inspirada de oxigênio (OR 0,99; IC 95% 0,98–0,99) poderiam ser fatores de proteção.

**Conclusões:**

Documentou-se alta frequência de LRA em pacientes com COVID-19, com diversos preditores: idade, sexo masculino, DM, DRC, PCR, admissão em UTI e suporte vasopressor. LRA ocorreu mais frequentemente em pacientes com maior gravidade da doença e associou-se a maior mortalidade e piores desfechos.

## Introduction

The first case of SARS-CoV-2 infection was reported in 2019, leading to a rapid increase in cases across several countries, and it was declared a pandemic in March 2020^1,2^. COVID-19 has caused great impact worldwide in terms of morbidity and mortality, costs to the healthcare system, and economic burden to affected countries^3,4^. It was initially considered a pulmonary disease, but over time, more was learned about its behavior, and it has been found that it could affect other organs, including the kidneys. Renal involvement by COVID-19 has been frequently reported in the medical literature and can manifest with hematuria, proteinuria, and/or renal dysfunction with acute kidney injury (AKI)^5^. The frequency of renal involvement varies in published studies; however, it has been reported in more than 20% of hospitalized patients and more than 50% in the intensive care unit (ICU) patients^6^. Multiple mechanisms are involved in the pathophysiology of AKI associated with COVID-19, such as viral kidney tropism and direct damage, inflammatory involvement, immune dysfunction, coagulopathy, endothelial dysfunction, complement activation, in addition to factors involved in the development of AKI in critically ill patients and their interventions^7^. AKI has been considered an independent risk factor for mortality in patients with COVID-19, reported in 35 to 80% of patients, being higher (75 to 90%) in patients requiring renal replacement therapy (RRT)^6-11^. Studies assessing AKI risk in COVID-19 patients have identified risk factors such as male sex, age, diabetes mellitus, heart disease, chronic kidney disease (CKD), black race, high non-renal SOFA score, severe COVID-19, invasive mechanical ventilation (IMV), and use of vasopressors^5,11–13^. In Colombia, the information available on the subject is limited. Considering the association of AKI with worse prognosis, it is important to stratify the risk of AKI to initiate preventive measures and early therapeutic strategies that could improve patient outcomes.

This study aimed to identify risk factors associated with the development of AKI in hospitalized patients diagnosed with COVID-19 in an academic hospital in Bogotá, Colombia, from March 1 to December 31, 2020. The secondary objective was to characterize the population and describe outcomes such as RRT and death.

## Methods

This was a descriptive, retrospective, cross-sectional study with an analytical component conducted at the Hospital Militar Central, Bogotá, Colombia. Initially, 1,019 patients were selected as they met the inclusion criteria of the study (adults aged 18 years or older with a diagnosis of COVID-19 by RT-PCR or antigen test, with length of hospital stay of more than 48 hours). A total of 934 patients were included in the analysis, while 22 were excluded due to 5D CKD, renal transplantation history, and pregnancy and 63 were excluded because of lack of information to evaluate the diagnosis of AKI.

AKI was defined according to the serum creatinine elevation criteria proposed by the 2012 KDIGO AKI guidelines (creatinine increase greater than or equal to 0.3 mg/dL within 48 hours or greater than or equal to 1.5 times from baseline within the last 7 days)^14^. The KDIGO classification was used to define the severity of AKI (1: creatinine increase of 1.5–1.9 times from baseline or greater than or equal to 0.3 mg/dL; 2: increase of 2–5 times; 3: increase greater than or equal to 3 times or increase in creatinine to greater than or equal to 4 mg/dL or onset of RRT)^14^. The criterion of decreased urine output was not included in the definition or severity of AKI because this information was not available in the patient’s clinical history.

For each patient, the average creatinine from days 8 to 365 before admission was calculated, and the lowest creatinine in the 7 days before admission was determined. The lowest creatinine value between these two parameters was defined as the baseline creatinine. If this information was not available, the lowest creatinine value during hospitalization (excluding the period in which the patient was on RRT) was taken as the reference creatinine.

Following approval by the ethics and research committee of the Hospital Militar Central, a database was created in an Excel spreadsheet, 2019 version, in which the information obtained from the patient’s electronic medical records was entered and then exported to the SPSS 21 statistical package for analysis. The variables recorded were demographic characteristics, history, clinical characteristics, baseline or reference creatinine, laboratory results of the first 72 hours of hospitalization, maximum and/or minimum value of some laboratory tests, and outcomes. Information about comorbidities was obtained from the review of medical records. Some variables had random missing data; however, no imputation by mean/median was performed to avoid altering the variable’s distribution. Due to the absence of linearity or correlation between some of these variables with missing data, no multiple or regression imputation was carried out. Instead, it was decided to conduct pairwise deletion.

An exploratory analysis of the data was initially performed. Quantitative variables were described as medians and interquartile ranges. Qualitative variables (nominal or ordinal) were described as absolute frequencies and relative percentages. For the univariate analysis, dummy variables were created according to cut-off points found in the medical literature, and subsequently in the bivariate analysis, the comparison of quantitative and qualitative variables was made between groups defined by the presence or absence of AKI in terms of socio-demographic characteristics, comorbidities, treatments received, baseline clinical-paraclinical status, and during follow-up, using the mood test for the comparison of medians and Pearson’s chi-square test or Fisher’s exact test for categorical variables. The magnitude and strength of the association were then determined by calculating odds ratios (OR) and their respective 95% confidence intervals (95% CI). Finally, we stratified by other variables using a multivariate logistic regression model, analyzing possible modifying or confounding variables of the measure of association. A p-value less than 0.05 was considered statistically significant.

To establish the risk factors associated with the development of AKI and RRT requirement, multivariate analysis was performed with binary logistic regression. Assumptions for the variables included in the model were verified, highlighting the fulfillment of error independence with a Durbin-Watson statistic ranging between 2 and 3. Additionally, the absence of multicollinearity was confirmed, with a variance inflation factor (VIF) statistic lower than 10 for each variable within the model. Variables of interest and with statistically significant differences were included in the bivariate analysis. Additionally, the calibration test (Hosmer and Lemeshow p = 0.022) was performed, and variables that affected the total number of cases to be analyzed due to low representation were excluded (n = 894, missing cases: 40 for development of AKI and n = 383, missing cases: 18 for RRT requirement), obtaining a Nagelkerke’s R-squared of 0.35.

## Results

The population analyzed had a median age of 62 years; most were men (68.6%), and the most frequent comorbidities were arterial hypertension (HTN) (45.2%), diabetes mellitus (DM) (19.4%), and obesity (14.6%). Only 7.3% of the patients had a history of CKD. About one-third of the patients were being treated with angiotensin-converting enzyme inhibitors (ACE inhibitors) and/or angiotensin II receptor blockers (ARB II). A total of 401 of the 934 patients (42.93%) developed AKI (60.59% KDIGO1, 17.95% KDIGO2 and 21.44% KDIGO3). Patients with AKI were significantly older than those who did not have this outcome; additionally, 60.09% of patients with AKI were 65 years of age or older compared to 35.83% of the group without AKI (p < 0.001). In patients with AKI, there was a higher proportion of male patients and comorbidities in terms of HTN, DM, ischemic heart disease, neoplasia, atherosclerotic vascular disease, CKD, and a greater frequency of treatment with ACEI and/or ARBs (Table 1).

**Table 1:** Baseline characteristics of hospitalized patients with COVID-19

Characteristic	Total cohort (n = 934)	AKI (n = 401)	No AKI (n = 533)	p value
Age – years, median (IQR)	63 (49–76)	70 (57–80)	58 (43–70)	<0.001
Sex – n (%)				
Male	641 (68.60)	307 (76.55)	334 (62.66)	<0.001
Female	292 (31.40)	94 (23.44)	199 (37.33)	
Comorbidities – n (%)				
HTN	422 (45.20)	228 (56.85)	194 (36.39)	<0.001
DM	181 (19.40)	108 (26.93)	73 (13.69)	<0.001
Obesity	136 (14.60)	60 (14.96)	76 (14.25)	0.76
COPD	94 (10.10)	45 (11.22)	49 (9.19)	0.31
Ischemic cardiopathy	85 (9.10)	51 (12.71)	34 (6.37)	0.001
Smoking	84 (8.99)	40 (9.97)	44 (8.25)	0.36
Neoplasm	76 (8.10)	45 (11.22)	31 (5.81)	0.003
Atherosclerotic vascular disease	74 (7.90)	50 (12.46)	24 (4.50)	<0.001
CKD	68 (7.30)	49 (12.21)	19 (3.56)	<0.001
Autoimmune disease	33 (3.50)	17 (4.23)	16 (3.00)	0.31
OSA	49 (5.20)	29 (7.23)	20 (3.75)	0.018
Asthma	18 (1.90)	6 (1.49)	12 (2.25)	0.4
HIV infection	5 (0.50)	4 (0.99)	1 (0.18)	0.09
ACE inhibitors and/or ARB	333 (35.70)	177 (44.13)	156 (29.26)	<0.001

HTN: Arterial hypertension, DM: Diabetes mellitus, COPD: Chronic obstructive pulmonary disease, CKD: Chronic kidney disease, OSA: Obstructive sleep apnea, HIV: Human immunodeficiency virus, AGE: Angiotensin converting enzyme, ARA: Angiotensin receptor blockers.

Some patients (33.2%) had a confirmed diagnosis of SARS-CoV2 infection on admission. In 88.9% of the remaining patients, infection was suspected on admission. Most patients reported respiratory symptoms on admission, and a smaller proportion reported fever and gastrointestinal symptoms. Treatment with dexamethasone was given to 96.5% of patients (Table 2). Table 2 also describes the laboratory results at hospital admission. Of note is the documentation of elevated lactate dehydrogenase (LDH), lactate, CRP, and D-dimer and decreased arterial partial pressure of oxygen/inspired oxygen fraction index (PaFiO_2_). Median values of creatinine and blood urea nitrogen (BUN) were normal. Patients with AKI had higher counts of leukocyte and neutrophils and higher levels of creatinine, LDH, CRP, D-dimer, troponin, and lactate than patients without AKI. Lymphocytes, platelets, arterial oxygen pressure (PO_2_), bicarbonate, and the PaFiO_2_ index were statistically lower in the group with AKI. The evolution of the laboratory tests during hospitalization showed that patients with AKI had higher peak values of leukocytes (15,910 vs 11,060, p < 0.001), neutrophils (13,840 vs 8,990, p < 0.001), creatinine (1.35 vs 0.92, p < 0.001), BUN (35.7 vs 19.4, p < 0.001), LDH (475 vs 330, p < 0.001), ferritin (19.5 vs 11.7, p < 0.001), CRP (22.7 vs 11.6, p < 0.001), troponin (25.6 vs 7.6, p < 0.001), D-dimer (2.6 vs 1.1, p < 0.001), PCO_2_ (49.1 vs 37.2, p < 0.001), and lactate (3.8 vs 3.1, p < 0.001) and lower minimum values of lymphocytes (440 vs 720, p < 0.001) and pH (7.30 vs 7.36, p < 0.001). Categorical analysis of some laboratory variables also showed that the AKI group had a higher percentage of hematological alterations, acid-base balance, hyperlactatemia, hypoxemia, and elevated inflammatory markers, consistent with the previous results (data not shown).

**Table 2:** Clinical characteristics and laboratory results at admission

Characteristic	Total cohort (n = 934)	AKI (n = 401)	No AKI (n = 533)	p value
Symptoms – n (%)				
Respiratory	861 (92.18)	359 (89.52)	502 (94.18)	0.009
Gastrointestinal	240 (25.69)	102 (25.43)	138 (25.89)	0.87
Fever	274 (29.33)	120 (29.92)	154 (28.89)	0.73
SARS-CoV-2 diagnostic test – n (%)				
RT-PCR	912 (97.64)	388 (96.75)	524 (98.31)	–
Antigen	22 (2.35)	13 (3.241)	9 (1.68)	–
NEWS2 score equal to or greater than 5 – n (%)	542 (68.17)	264 (78.1)	278 (60.83)	<0.001
Treatment – n (%)				
Dexamethasone	901 (96.50)	391 (97.50)	510 (95.68)	0.13
Ivermectin	679 (72.70)	300 (74.81)	379 (71.10)	0.21
Chloroquine or hydroxichloroquine	3 (0.30)	1 (0.24)	3 (0.37)	0.73
Vancomycin	60 (6.40)	52 (12.96)	8 (1.50)	<0.001
Blood count, median (IQR)				
WBC – n/mm^3^	7,850 (5,805–10,805)	8,330 (6,115–11,817)	7,530 (5,500–10,280)	<0.001
Neutrophils – n/mm^3^	5,870 (4,000–8,715)	6,350 (4,220–9,502)	5,610 (3,800–8,300)	<0.001
Lymphocytes – n/mm^3^	1,080 (760–1,450)	1,020 (712–1,390)	1,120 (790–1,485)	0.013
Hemoglobin – gr/dL	14.90 (13.70–16.17)	14.90 (13.62–16.30)	14.90 (13.72–16.10)	0.901
Platelets – n/mm^3^	224,000 (17,000–277,000)	209,000 (161,000–267,000)	233,000 (185,000–282,500)	<0.001
Creatinine – mg/dL	0.93 (0.78–1.11)	1.07 (0.89–1.33)	0.87 (0.73–0.99)	<0.001
Blood urea nitrogen- mg/dL	15.65 (11.40–21.12)	19.30 (14.55–28.10)	13.60 (10.50–17.55)	<0.001
LDH – U/L	314 (253–410)	353 (274–480)	297 (245–382)	<0.001
Ferritin – ng/mL	901 (455–1,596)	933 (523–1,711)	880 (428–1,505)	0.073
CRP – mg/dL	9.98 (4.48–18.67)	12 (5.55–22.71)	8.91 (4.03–16.45)	<0.001
D dimer – µg/mL	0.94 (0.60–1.63)	1.18 (0.70–2.22)	0.86 (0.55–1.37)	<0.001
Troponin – ng/mL	8.40 (5.20–15.52)	12.20 (7.50–22.60)	6.40 (4.60–10.20)	<0.001
Arterial gases				<0.001
pH	7.41 (7.38–7.44)	7.41 (7.37–7.43)	7.42 (7.39–7.44)	0.101
PCO_2_ – mmHg	32.80 (29.80–36.20)	32.50 (29.32–32.97)	33.05 (30–36.30)	0.019
PO_2_ – mmHg	64.05 (54.40–74.40)	62.95 (52.82–74.80)	65 (56.20–74.20)	<0.001
HCO_3_ – mEq/L	20.70 (19.10–22.30)	20.10 (18.30–22.17)	21 (19.60–22.40)	<0.001
PaOFiO_2_	245.34 (185.66–296.43)	221.79 (163.13–286.43)	248 (201–303)	
Lactate – mmol/L	2.16 (1.75–2.79)	2.24 (1.82–2.89)	2.08 (1.68–2.66)	0.001

RT-PCR: Real-time reverse transcription–polymerase chain reaction, NEWS2: National Early Warning Score, WBC: White blood count, LDH: Lactate deshydrogenase, CRP: C reactive protein, PCO_2_: Carbon dioxide arterial pressure, PO_2_: Oxygen arterial pressure, HCO_3_: Bicarbonate, PaFiO_2_: Partial pressure arterial oxygen/inspired oxygen fraction.

The median duration of hospitalization was 11 days; 31.37% required admission to the ICU, 21.84%, treatment with vasopressors, 5.24%, inotropic drugs, and 23.23%, IMV. The main ICU admission indication was ventilatory failure (91.8%). In-hospital mortality was 23.2% (217 patients). Patients with AKI had a longer length of hospital stay (16 days vs. 9 days), higher mortality (44.6% vs. 7.1%), ICU admission (60.8% vs. 9.1%), IMV (49.8% vs. 3.1%), vasopressor support (47.8% vs. 2.2%), inotropic support (11.4% vs. 0.5%), and more frequent bacterial coinfection (24.4% vs. 4.8%), with p-values less than 0.001 (Table 3). Of those patients who required IMV and had AKI, 60% (120/200) developed AKI after the initiation of ventilation. Concerning patients who required vasopressor support and had AKI, 55.2% (106/192) developed AKI after the initiation of medication.

**Table 3:** General outcomes

Outcome	Total cohort (n = 934)	AKI (n = 401)	No AKI (n = 533)	p value
Hospital length of stay – days, median (IQR)	11 (8–18)	16 (11–24.50)	9 (7–12)	<0.001
ICU – n (%)	293 (31.37)	244 (60.84)	49 (9.19)	<0.001
Vasopressors – n (%)	204 (21.84)	192 (47.88)	12 (2.25)	<0.001
Inotropes – n (%)	49 (5.24)	46 (11.47)	3 (0.56)	<0.001
IMV – n (%)	217 (23.23)	200 (49.87)	17 (3.18)	<0.001
Bacterial coinfection – n (%)	124 (13.30)	98 (24.4)	26 (4.87)	<0.001
In-hospital death – n (%)	217 (23.20)	179 (44.63)	38 (7.12)	<0.001
Hospital readmission (30 days) – n (%)	39 (4.17)	17 (4.23)	21 (3.93)	0.82

ICU: Intensive care unit, IMV: Invasive mechanical ventilation.

Regarding renal outcomes, a significant percentage (43.89%) had AKI at admission. Mortality in the group that developed AKI was 44.63%, significantly higher than in the group that did not experience AKI (7.12%, p < 0.001). Forty patients (4.28% of the total population and 9.9% of the AKI population) required RRT, 75% with continuous renal replacement therapy (CRRT), 20% with intermittent hemodialysis, and 5% with both modalities. Femoral vein catheter was the most frequent vascular access (60%). More than half of the patients required RRT due to volume overload and/or oliguria/anuria (52.5%). Other less frequent indications were metabolic acidosis (17.5%), uremia (15%), and hyperkalemia (15%). The median duration of RRT was 4 days (RIQ 2-9). The median time of RRT onset was one day after AKI diagnosis (IQR 0-3). Out of the 40 patients who required RRT, 36 died (90%), discontinuation of therapy was achieved in three patients, and one patient was discharged on dialysis. The logistic model that evaluated the risk factors for RRT showed that the variables of vasopressor support (OR 15.97; 95% CI 6.75–37.8, p < 0.001), D-dimer at admission greater than 1 μg/mL (OR 5.35; 95% CI 2.1–13.61, p < 0.001), and lactate at admission greater than 2 mmol/L (OR3.07; 95% CI 1.32–7.12, p = 0.009) impacted the probability of RRT in patients with AKI and COVID-19.

The logistic model that evaluated the risk factors for AKI showed that the variables age (OR 1.03; 95% CI 1.02–1.04), male sex (OR 2.13; 95% CI 1.49–3.04), DM (OR 1.55; 95% CI 1.04–2.32), CKD (OR 2.07; 95% CI 1.06–4.04), CRP at admission (OR 1.02; 95% CI 1.00-1.03), ICU admission (OR 1.81; 95% CI 1.04–3.16), and vasopressor support (OR 7.46; 95% CI 3.34–16.64) impacted the probability of developing AKI in the population and that bicarbonate (OR 0.89; 95% CI 0.84–0.94) and PaFiO_2_ (OR 0.99; 95% CI 0.98–0.99) were protective factors (Table 4).

**Table 4:** Odds ratios of AKI development in the unadjusted and adjusted model

Total cohort (n = 934)	Unadjusted		Adjusted	
	OR (95% CI)	P value	OR (95% CI)	p value
Age	1.36 (1.02–1.04)	<0.001	1.03 (1.02–1.04)	<0.001
Sex	1.94 (1.45–2.60)	<0.001	2.13 (1.49–3.04)	<0.001
Comorbidities				
Arterial hypertension	2.30 (1.76–3.00)	<0.001	–	–
Diabetes mellitus	2.32 (1.66–3.23)	<0.001	1.55 (1.04–2.32)	0.031
Obesity	1.05 (0.73–1.52)	0.763	–	–
COPD	1.24 (0.81–1.91)	0.308	–	–
Ischemic cardiopathy	2.13 (1.35–3.37)	<0.001	–	–
Smoking	1.23 (0.78–1.93)	0.363	–	–
Neoplasm	2.04 (1.27–3.29)	0.003	–	–
Atherosclerotic vascular disease	3.02 (1.82–5.00)	<0.001	–	–
CKD	3.76 (2.18–6.50)	<0.001	2.07 (1.06–4.04)	0.031
Autoimmune disease	1.43 (0.71–2.86)	0.311	–	–
OSA	2.00 (1.11–3.59)	0.018	–	–
Asthma	0.65 (0.24–1.77)	0.406	–	–
HIV infection	5.36 (0.59–48.14)	0.093	–	–
ACE inhibitors and/or ARB	1.91 (1.45–2.50)	<0.001	–	–
CRP – mg/dL	1.03 (1.01–1.04)	<0.001	1.02 (1.00–1.03)	0.006
Platelets – n/mm^3^	1.00 (1.00–1.00)	<0.001	1.00 (1.00–1.01)	0.01
HCO_3_ – mEq/L	0.87 (0.83–0.92)	<0.001	0.89 (0.84–0.94)	<0.001
PaFiO_2_	0.99 (0.99–0.99)	<0.001	0.99 (0.98–0.99)	0.014
ICU	5.18 (3.62–7.42)	<0.001	1.81 (1.04–3.16)	<0.001
Vasopressors	15.8 (8.5–29.18)	<0.001	7.46 (3.34–16.64)	<0.001
Inotropes	1.00 (0.99–1.01)	0.264	–	–
IMV	0.70 (0.48–1.02)	0.067	–	–
Age ≥ 65 years	2.69 (2.06–3.52)	<0.001	–	–
WBC ≥ 13000/mm^3^	1.87 (1.30–2.69)	<0.001	–	–
Neutrophils ≥ 10000/mm^3^	1.69 (1.21–2.35)	0.002	–	–
Lymphocytes < 1000/mm^3^	1.44 (1.11–1.87)	0.006	–	–
Platelets < 100000/mm^3^	2.55 (1.07–6.09)	0.028	–	–
HCO3 < or > 22 mEq/L	1.31 (1.01–1.71)	0.038	–	–
LDH ≥ 350 U/L	2.17 (1.65–2.86)	<0.001	–	–
CPR ≥ 10 mg/dL	1.54 (1.18–2.01)	0.001	–	–
Troponina ≥ 10 ng/mL	4.59 (3.43–6.14)	<0.001	–	–
pH < 7.35 or >7.45	1.13 (0.84–1.53)	0.398	–	–
PO_2_ < 60 mmHg	1.30 (1.00–1.70)	0.047	–	–
Lactate >2 mmol/L	1.52 (1.16–1.99)	0.002	–	–
PaFiO_2_ < 200	2.14 (1.61–2.84)	<0.001	–	–
PaFiO_2_ <100	2.75 (1.27–5.95)	0.007	–	–
D-dimer > 0.5 ug/mL	0.49 (0.33–0.72)	<0.001	–	–
Ferritine > 1000 ng/mL	1.25 (0.96–1.64)	0.092	–	–
NEWS ≥ 5	2.29 (1.67–3.16)	<0.001	–	–

COPD: Chronic obstructive pulmonary disease, CKD: Chronic kidney disease, OSA: Obstructive sleep apnea, HIV: Human immunodeficiency virus, ACE: Angiotensin converting enzyme, ARB: Angiotensin receptor blockers, CRP: C reactive protein, HCO_3_: bicarbonate, ICU: Intensive care unit, IMV: Invasive mechanical ventilation, LDH: Lactate dehydrogenase, WBC: White blood count, PO_2_: Oxygen arterial pressure, PaFiO_2_: Partial pressure arterial oxygen/inspired oxygen fraction, NEWS2: National Early Warning Score.

## Discussion

During hospitalization, 43.93% of the patients with COVID-19 developed AKI, indicating a high incidence of renal involvement. This rate was higher than that previously reported, particularly in Asia. Paek et al.^15^ reported a rate of AKI of 4% in 704 patients from Daegu, Korea. Similarly, Cheng et al.^8^ reported a rate of 5.1% in 701 patients from Wugan, China. A metanalysis that analyzed the incidence by continents documented lower rates in Asia (6.9%) compared to Europe (22.9%) and North America (34.6%)^16^. Three large COVID-19 population studies conducted in New York, United States, reported AKI rates from 36.6% to 46%, similar to the results of the present study^5,11,17^. It has been suggested that these differences may be due to a higher proportion of patients with comorbidities in studies from western countries^16^. In Latin America, studies have reported incidences up to 58.6%; however, they included only critically ill patients or patients with severe COVID-19, unlike this study that also included patients in the general ward^18-20^.

In comparison with the population that did not develop AKI, the AKI group had a higher frequency of comorbidities such as HTN, DM, neoplasia, and cardiovascular disease. This is consistent with what has been reported in the literature, where these diseases are prevalent and have been associated with disease severity and outcomes^21,22,23,24,25^. CKD was more frequently observed in the population with AKI; however, this condition was probably underestimated because the glomerular filtration rate was not estimated with the baseline creatinine of the patients. Similar findings were reported in two studies conducted in New York, where CKD was evaluated in the same way. They reported a prevalence of 2.9% and 11% in the COVID-19 population, also showing differences between groups^11,17^. On the other hand, obesity, which has been linked with severe COVID-19, could have been underestimated since the body mass index of the patients was not calculated, considering only the diagnosis recorded in the medical history^26^. No differences in the frequency of obesity were found, nor obesity was identified as a risk factor for AKI.

It is worth highlighting that about one-third of the patients already had a diagnosis of COVID-19 at the time of admission, and of those who did not have a diagnosis, 88.9% were suspected to be infected at their admission, suggesting that the laboratory tests taken in the first 72 hours could already reflect alterations associated with the infection. In these laboratory tests, patients with AKI more frequently presented hematological alterations, elevated inflammatory markers, acid-base balance disorders, and compromised oxygenation. Similar results were reported by Ng et al.^17^ in their study, in which COVID-19 patients with AKI had higher levels of leukocytes, CRP, D-dimer, and ferritin at admission compared to those without AKI. D-dimer, CRP, and ferritin levels were even higher in AKI patients requiring RRT compared to those who did not. These findings can be observed in COVID-19 patients due to the disease’s inflammatory component, compromised oxygenation, or, in some cases, tissue perfusion^27,28^.

In terms of outcomes, patients with AKI experienced a longer hospital stay, higher mortality, ICU admission, vasopressor and inotropic support, and ventilatory support compared to patients without AKI. Chan et al.^11^ reported similar results in a cohort of 3,993 hospitalized patients with COVID-19 in five centers in New York (AKI versus no AKI: hospital stay 10 vs 7 days, mortality 50% vs 8%, ICU admission 41% vs 11%, vasopressor support 43% vs 10%, IMV 44% vs 6%, with significant p-values). These results have also been reproduced by other authors^4,7^. Additionally, AKI has been described as an independent risk factor for mortality in COVID-19^29^. Another study conducted by our group on the same Colombian population documented the impact of AKI on mortality (OR 12.5; 95% CI 2.1–74.5, p = 0.005)^30^. These findings highlight the prognostic importance of AKI development in COVID-19 patients, contributing to severe outcomes and greater disease burden. They also underscore the relationship of AKI development in the context of inflammatory diseases and/or multiorgan dysfunction.

Almost 10% of AKI patients required RRT. RRT has been reported to be between 10% and 20% in hospitalized patients with AKI and COVID-19 and even higher depending on the studied population^5,11,17^. In a multicenter study evaluating 3,099 critically ill COVID-19 patients, Gupta et al.^31^ reported RRT requirement in 37.8% of AKI patients. The difference could be attributed to the fact that all patients were in ICU, while in the present study, 39.2% were hospitalized in the general ward.

The starting modality of RRT was CRRT in 75% of the patients; this was expected due to the severity of the disease and the critical condition of the patients, since all the patients who required RRT were in the ICU and 97.5% were on vasopressor support. The main indication for RRT was volume overload and/or oliguria/anuria. During hospitalization, peak creatinine and BUN values were not as high as expected (data not shown), which is consistent with the fact that hyperazotemia was not a frequent RRT indication. In addition, fluid accumulation increases total body water and alters serum creatinine volume distribution, which affects measured creatinine levels^32^. However, this study lacked information on the fluid balance of patients, so this assumption could not be confirmed.

Mortality is high in patients with RRT and COVID-19, reported up to 63.3% and 79.3%^17,31^. In this study, mortality was even higher at 90%. Because this was not an objective of the study, there is insufficient information available to conduct an analysis of mortality in patients with RRT. This would require collecting information on dialysis dosage, fluid balance, temporal CRRT suspension, and compliance with therapeutic goals. The median RRT initiation time was 1 day after AKI diagnosis, suggesting early intervention. However, it is important to consider that AKI diagnosis did not consider the urine output criteria, so AKI could have occurred earlier than considered, potentially affecting the interpretation of these data.

Finally, regarding risk factors associated with the development of AKI, the model was calibrated and had a statistically significant p-value, indicating model consistency. Age, male sex, ICU admission, history of DM, CKD, CRP, and vasopressor support were factors associated with AKI development. These have already been reported in the literature as risk factors for AKI in the COVID-19 population^12,13,33^.

CKD and AKI are interconnected syndromes. Patients with CKD are predisposed to AKI because they have lower renal functional reserve and have greater susceptibility to new renal injury^34^. An AKI event in patients with preexisting CKD could accelerate the underlying CKD, and in the COVID19 population, poor baseline renal function was found to be related with increased hospitalization, mortality rates, and RRT requirement^35^. Chan et al.^11^ reported that CKD is a risk factor for severe AKI in a population of 3,993 hospitalized patients with COVID-19 (OR 2.8, 95% CI 2.5–3.7). The data reported by Gupta et al.^31^ also support this finding and, on the other hand, document that age (older than 80 years) is a risk factor for the composite outcome of RRT and death (OR 3.69; 95% CI 2.33–5.82).

The role of inflammation in COVID-19 should be emphasized as it may be related to adverse outcomes. CRP is a marker of disease severity and magnitude of the inflammatory response. Smilowittz et al.^36^ reported that CRP is related to the development of AKI in COVID-19 patients (OR 2.11; CI 95% 1.76–2.52) and to venous thromboembolism, disease severity, and mortality. In the present study, CRP at hospital admission was associated with AKI development. Although peak levels of inflammatory markers were higher in patients with AKI than in those without AKI, these variables were not included in the analysis of AKI risk factors due to a lack of data on the temporal relationship between peak levels and AKI onset.

Bicarbonate and PaFiO_2_ are indicators of acid-base status and oxygenation, which are often altered in COVID-19 patients. Hypoxemia and acidosis can lead to dysregulation of the immune system and inflammatory reactions, potentially resulting in a cytokine storm^37^. While acidosis per se has not been documented as a cause of AKI, it is essential to note that acidosis is a marker of disease severity and that organ dysfunction can be associated with AKI development. There are several pathophysiological factors that explain the lung-kidney interaction, including alterations in blood gases due to hypoxemia, which reduces renal blood flow through increase in renal vascular resistance^38^. The result of the multivariate analysis suggests that bicarbonate and PaFiO_2_ could be potential protective factors against AKI, consistent with what was mentioned earlier.

Some studies have reported IMV as a risk factor for AKI in COVID-19^12,13^. Despite the fact that in this study AKI patients had a higher requirement for IMV and 60% of patients who required IMV and developed AKI did so after starting ventilation, IMV did not emerge as a risk factor in the described population.

The present study had several limitations. First, it was a retrospective study possibly generating information bias. Second, it was a single center study limiting its external validity. Third, baseline creatinine was not available for 91.76% of the patients. In this group, the outcome of AKI was evaluated by comparison with the reference creatinine, as described in the Methods section. Fourth, urine output was not included as a diagnostic criterion or as a measure of AKI severity. These last two points could have generated inconsistencies in the measurement of AKI outcome^30^.

## Conclusions

This study shows the behavior of COVID-19 in the Colombian population, and, as far as we know, there are no studies in our country with such a large population in a single center. The study results are consistent with those reported in the medical literature, highlighting the high frequency of AKI and it’s prognostic impact. The presence of AKI in patients with severe disease parameters is noteworthy. The risk factors associated with development of AKI are related to male sex, hemodynamic impairment and comorbidities, including DM and CKD.

Risk stratification based on previously described parameters will be important to tailor monitoring and initiate strategies for patients who will benefit from intervention. Additionally, it should raise awareness for post-COVID-19 follow-up, as AKI on CKD could impact the baseline renal function of patients.
